# Diversity in Sensing and Signaling of Bacterial Sensor Histidine Kinases

**DOI:** 10.3390/biom11101524

**Published:** 2021-10-15

**Authors:** Eiji Ishii, Yoko Eguchi

**Affiliations:** 1 Department of Bacterial Infections, Research Institute for Microbial Diseases, Osaka University, Osaka 565-0871, Japan; eishii@biken.osaka-u.ac.jp; 2 Faculty of Biology-Oriented Science and Technology, Kindai University, Kinokawa 649-6493, Japan

**Keywords:** two-component signal transduction system, histidine kinase, autophosphorylation, phosphatase, ligand binding, PAS, intramembrane sensing, environmental signal sensing

## Abstract

Two-component signal transduction systems (TCSs) are widely conserved in bacteria to respond to and adapt to the changing environment. Since TCSs are also involved in controlling the expression of virulence, biofilm formation, quorum sensing, and antimicrobial resistance in pathogens, they serve as candidates for novel drug targets. TCSs consist of a sensor histidine kinase (HK) and its cognate response regulator (RR). Upon perception of a signal, HKs autophosphorylate their conserved histidine residues, followed by phosphotransfer to their partner RRs. The phosphorylated RRs mostly function as transcriptional regulators and control the expression of genes necessary for stress response. HKs sense their specific signals not only in their extracytoplasmic sensor domain but also in their cytoplasmic and transmembrane domains. The signals are sensed either directly or indirectly via cofactors and accessory proteins. Accumulating evidence shows that a single HK can sense and respond to multiple signals in different domains. The underlying molecular mechanisms of how HK activity is controlled by these signals have been extensively studied both biochemically and structurally. In this article, we introduce the wide diversity of signal perception in different domains of HKs, together with their recently clarified structures and molecular mechanisms.

## 1. Introduction

Microorganisms employ various signaling systems to sense and adapt to changing environmental conditions. The two-component signal transduction system (TCS) is one of the major mechanisms of signal transfer in prokaryotes and is present in most bacteria and many archaea [1,2]. Although smaller in number, some TCSs are also present in eukaryotes, such as fungi and plants, but none are found in mammals. TCSs control various aspects of bacterial cell physiology, such as resistance to physical and chemical stresses, cell division, biofilm formation, quorum sensing, and sporulation. A large number of TCS research studies have been conducted on bacterial pathogens because TCSs often control the expression of virulence factors, including colonization, toxin production, toxin secretion, invasion, survival in host cells, and antimicrobial resistance [3,4,5]. Hence, together with their absence in mammalian cells, TCSs are recognized as potential targets for antimicrobial drug design [3,4,6]. Information on 164,651 TCS proteins from 2758 sequenced prokaryote genomes can be accessed at the P2CS (prokaryotic two-component system) database (http://www.p2cs.org, accessed on 1 September 2021) [7]. A comprehensive classification of microbial signal transduction systems, including TCSs, can also be found in the MiST3.0 (microbial signal transduction) database (http://mistdb.com, accessed on 1 September 2021) [8].

**Figure 1:**
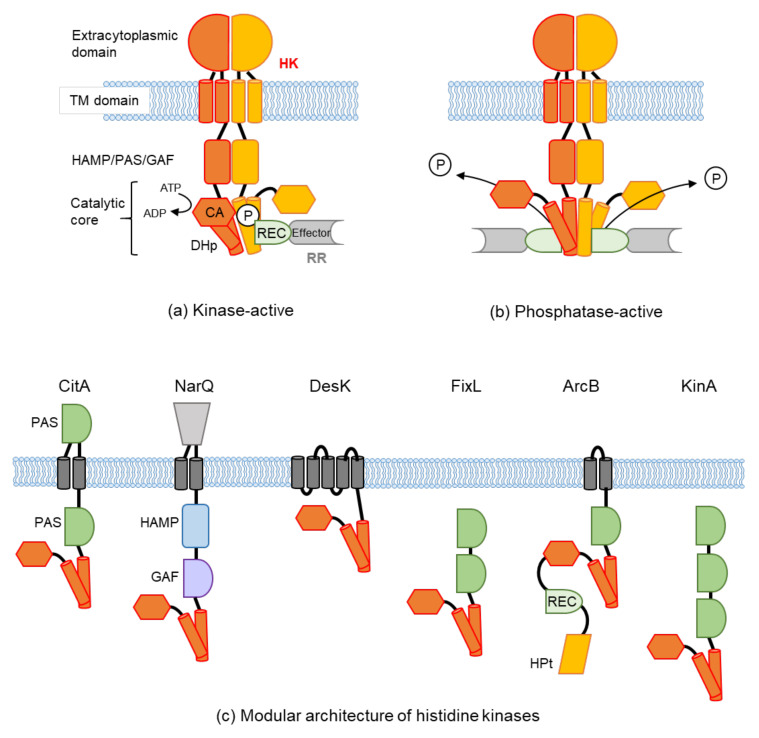
Schematic representation of a prototypical sensor histidine kinase (HK) and its variants. (**a**) In a prototypical HK, a kinase-activating signal perceived by the extracytoplasmic domain is transduced across the membrane via the transmembrane (TM) domain, transmitted through the intracellular signal domains (HAMP, a domain found in histidine kinases, adenylyl cyclases, methyl binding proteins, and phosphatases; PAS, Per-ARNT-Sim; or GAF, a domain found in cGMP-specific phosphodiesterase, adenylyl cyclases, and FhlA), and eventually to the catalytic core, consisting of DHp (dimerization and histidine phosphorylation) and CA (catalytic/ATP binding) domains. The catalytic core adopts an asymmetric kinase-active conformation with ATP binding to the CA domain and autophosphorylating the conserved His residue in the DHp domain. The phosphate moiety (P) is then transferred to the Asp residue in the receiver (REC) domain of its partner response regulator (RR). The phosphorylated RR triggers downstream responses through its effector domain. (**b**) When the extracytoplasmic domain perceives a repressive signal, this signal is also transduced to the catalytic core. The catalytic core changes into a symmetric phosphatase-active conformation. The phosphorylated RRs are dephosphorylated, which turns off the system. (**c**) Variation in the modular architecture of various HKs. HKs normally exist as dimers but are shown as monomers for simplification. HKs can either reside in the membrane or in the cytoplasm without TM helices. Numbers and locations of signal domains such as HAMP, PAS, or GAF vary among HKs. In hybrid HKs such as ArcB, the receiver (REC) and histidine phosphotransfer (HPt) domains are fused to the C-terminal end of the CA domain. The phosphate moiety is relayed from the DHp domain to the REC and HPt domains before being transferred to the RR.

TCSs are generally composed of a sensor histidine kinase (HK) and its partner response regulator (RR). HKs are mostly homodimeric and are located either at the membrane or inside the cell. Approximately 83% of HKs contain transmembrane regions [9]. A prototypical HK (Figure 1a) is composed of an extracytoplasmic or periplasmic sensor domain, transmembrane (TM) domain, usually one or more intracellular signal domains (HAMP, a domain found in histidine kinases, adenylyl cyclases, methyl binding proteins, and phosphatases; PAS, Per-ARNT-Sim; or GAF, a domain found in cGMP-specific phosphodiesterase, adenylyl cyclases, and FhlA), and a catalytic core (or kinase domain) that consists of a DHp (dimerization and histidine phosphorylation) domain and a CA (catalytic/ATP binding) domain. Upon signal perception in the sensor domain, the signal is transduced across the membrane via the TM domain, transmitted through the intracellular signal domains, and eventually to the catalytic core. At the catalytic core, ATP binds to the CA domain and autophosphorylates the conserved His residue in the DHp domain. This is followed by the transfer of the phosphate moiety to the conserved Asp residue in the receiver (REC) domain of the partner RR. The phosphorylated RR triggers downstream responses most frequently by binding to DNA for transcriptional control of its target gene. In some cases, RRs bind to RNA or proteins, or they exert enzymatic activity [10].

In addition to their autokinase activity, most HKs can dephosphorylate their phosphorylated RR partners (Figure 1b). This controls the level of phosphorylated RRs, which adjust the cellular response against the perceived signal. The kinase-active state is often referred to as the ON state or HK activation, and the phosphatase-active state is referred to as the OFF state or HK inactivation (or repression of HK). Biochemical, structural, and molecular dynamics (MD) simulation studies of each domain or a set of domains have elucidated the molecular mechanism of signal transduction within HKs (reviewed in [10,11,12,13,14,15,16]). The structural data of the catalytic core or in the complex with the REC domain of its partner RR showed a symmetrical and static phosphatase state (Figure 1b) and the asymmetrical and dynamic kinase state (Figure 1a) of HKs. Furthermore, the structures suggest that autophosphorylation occurs in one protomer at a time [14].

How the signal received at the periplasmic (or extracytoplasmic) region is transmitted to the cytoplasmic domain through the transmembrane region has been of great interest. Although crystal structures of a full-length membrane-bound HK are still not available, Gushchin et al. determined the structures of a set of the periplasmic sensor, TM, and cytoplasmic HAMP domains of the *Escherichia coli* nitrate/nitrite-sensing NarQ HK in ligand-bound and ligand-free states [17]. Comparison of the two states revealed that ligand binding induced piston-like displacements of the TM helices, and this piston-like movement was amplified and converted into a helical rotation at the cytoplasmic HAMP domain. Transmembrane signaling from the periplasmic region to the cytoplasm is further discussed in a later section of this article.

Variations in the modular architecture of the representative HKs are shown in Figure 1c. Signal recognition domains can be found both inside and outside the cell, and the number and combination of these modules differ among HKs. In contrast with the variation in signal recognition and transduction modules, the catalytic core of the DHp and CA domains is well conserved among HKs. Hybrid HKs, such as ArcB and BvgS, have receiver (REC) and histidine phosphotransfer (HPt) domains in addition to the DHp and CA domains. In these HKs, the phosphate moiety is relayed from the DHp domain to the REC and HPt domains before being transferred to the partner RR. HK activity is modulated not only by the signal perceived at its signal recognition domain but also by the cytoplasmic signal transduction domains and the catalytic core (reviewed in [18,19]). The number of identified signals recognized by HKs is continuously growing, and the variation in how the signals are perceived is expanding. Furthermore, a single HK can sense multiple signals in different domains and can integrate the information to fine-tune its response.

In the present article, we introduce the wide diversity of signal perception by HKs, mostly focusing on recent examples where the molecular mechanisms of signal perception have been proposed. We begin with signal sensing at the extracytoplasmic or periplasmic sensor domain, then at the TM domain, and finally at the cytoplasmic domain. Signals may be direct binding of ligands, indirect binding of ligands via accessory proteins and cofactors, or binding of proteins and second messengers. The direct sensing of physical and chemical signals—such as temperature, osmotic pressure, redox, and pH by HKs—is also known. Readers will notice that the same HK appears in various sections, indicating that a HK can sense different signals in various domains and somehow integrate the information.

## 2. Extracellular Sensing

The extracellular or periplasmic region located at the N-terminus of many HKs is called the sensor domain, and it is responsible for sensing environmental signals and transmitting the information to the cytoplasmic kinase domain in various HKs [11,18,20,21]. Since the structures of the periplasmic region of CitA and DcuS [22,23] were clarified, many structures of extracellular sensors that can sense various signals have been reported (Table 1). In this section, we introduce the representative structures of periplasmic sensor domains and the signals (ligands) that bind to them. Some of the latest structures are also described.

### 2.1. Structural Features of Periplasm Sensors and Ligand Binding

#### 2.1.1. Sensing by a PAS-Like Domain

The CitA HK of Klebsiella pneumoniae, paired with its response regulator CitB, senses environmental citrate and induces the expression of genes involved in citrate fermentation. CitA is the structure of the periplasmic region of CitA HK. This is one of the first structures of an extracellular domain of a HK with a PAS fold. The first structure of a sensor domain with a PAS fold in a HK was found in the cytoplasmic region of FixL HK (Figure 1c, more details are provided in Section 4) [24]. The PAS domain acts as a diverse sensor and interaction module for signal transduction. The amino acid sequence of the PAS domain is not well conserved, making it difficult to predict PAS domains from amino acid sequences. In contrast, the secondary structures of PAS domains are highly conserved, with multiple β-sheets in the core and several α-helices [16,25,26]. Most PAS domains in bacteria are found in the cytoplasm (for cytoplasmic sensing of HKs; more details are provided in Section 4) [24]. It was only after the determination of the periplasmic PAS structure of CitA and DcuS that the periplasmic PAS fold could be predicted from the secondary structure. The periplasmic PAS of HK is called the PDC (PhoQ-DcuS-CitA) region, which includes PhoQ HK [27,28], the structure of which was later solved (PAS of the periplasmic sensor is hereafter referred to as PDC) [29].

Generally, a ligand binds to the well-conserved cleft formed by the inner β-sheet and α-helices of the PAS domain [16]. In CitA, citrate fits into this conserved cleft-like region [22,30]. The sensor domains of DcuS HK (RR: DcuR) of *E. coli* and DctB HK (RR: DctD) of *Rhizobium* are members of the same PDC family and can sense C4 dicarboxylates (such as fumarate, succinate, malate, and tartrate) in the PDC. DcuS has a single PDC, while DctB has two PDCs in tandem: a membrane-distal PDC and a membrane-proximal PDC [23,29]. When these three PDCs are compared with that of CitA, the PDC of DcuS and the membrane-distal PDC of DctB both show a similar structure to that of CitA. The (malate and succinate) ligand-binding positions of DcuS and the distal PDC of DctB are in the same cleft-forming region as the ligand-binding region of CitA. However, the function of the membrane-proximal PDC of DctB remains unclear. Double PDCs were observed not only in DctB but also in other HK periplasmic structures (Table 1). KinD of *Bacillus subtilis*, which is involved in sporulation and biofilm formation, also has a double PDC structure and, similar to DctB, has a (pyruvate) ligand-binding site in the conserved cleft structure of the membrane-distal PDC [31]. Ligand binding to a highly conserved structure may seem to lose specificity; however, as previously mentioned, amino acid sequence homology among PDCs is low, and the specificity of the PDC ligand is maintained by the amino acid composition in the cleft region. In fact, the ligand and specified amino acids in the cleft form salt bridges. The importance of the character and steric arrangement of individual amino acids has been demonstrated by structural insight and mutagenesis analysis [31].

**Table 1:** Signal Sensing by Extracellular domain of HK.

Sensor	PDB	Fold Type	Ligand *	Organism	Ref.
***PDC structures***					
CitA	2V9A, 5FQ1, 1P0Z, 2J80	PDC	citrate	*K. pneumoniae* *Geobacillus thermodenitrificans*	[22,32,33]
DcuS	1OJG	PDC	malate, oxygen	*E. coli*	[29]
PhoQ	3BQ8, 1YAX, 6A8V	PDC	Ni^2+^, Mg^2+^, Ca^2+^, SafA	*E. coli* *Salmonella typhimurium*	[27,28,34]
PhoR	3CWF	PDC		*Bacillus subtilis*	[35]
EnvZ	5XGA	PDC	CHAPS, MzrA	*E. coli*	[36,37]
CusS	5KU5	PDC	Ag(I)	*E. coli*	[38]
BaeS	5WVN 5WVM	PDC	indole	*E. coli*	[39]
DctB	3E4O, 3E4Q, 3E4P, 3BY9	double PDC	succinate, Ca^2+^, oxygen	*Sinorhizobium meliloti* *V. cholerae*	[29,40]
mmHK1S-Z2	3LI9, 3LIA, 3LI8	double PDC	bistris	*Methanosarcina mazei*	[41]
mmHK1S-Z3	3LIB	double PDC		*M. mazei*	[41]
soHK1S-Z6	3LIC	double PDC	ethylene glycol	*Shewanella oneidensis*	[41]
vpHK1S-Z8	3LIE 3LID	double PDC	phosphate	*V. parahaemolyticus*	[41]
rpHK1S-Z16	3LIF	double PDC	2 methyl-2,4- pentanediolMPD	*Rhodopseudomonas palustris*	[41]
HptSA	6LKG, 6LKI	double PDC	G6P	*S. aureus*	[42]
LuxQP	2HJE, 2HJ9	double PDC	AI-2	*Vibrio harveyi*	[32,43]
KinD	4JGP, 4JGO, 4JGQ,4JGR	double PDC	pyruvate	*B. subtilis*	[31]
***All helical structures***					
NarX	3EZH	all α-helix	nitrate ion	*E. coli*	[44]
NarQ	5IJI, 5JGP, 5JEF, 5JEQ, 6XYN,6YUE	all α-helix	nitrate ion	*E. coli*	[17,45,46,47]
TorS	3I9W, 3I9Y, 3O1J, 3O1I, 3OIH	all α-helix	TorT, TMAO	*E. coli*	[48,49]
KinB	3KKB, 3L34, 4LLE, 4LLC	all α-helix	phosphate ion	*P. aeruginosa*	[50]
Adeh_2942	4K0D	all α-helix	acetate	*Anaeromyxobacter dehalogenans*	[51]
***Other structures***					
BvgS	4Q0C, 3MPL, 3MPK	double VFT	nicotine	*B. pertussis*	[52,53]
BT4663	4A2M, 4A2L	Y_Y_Y domain		*Bacteroides thetaiotaomicron*	[54]
VbrK	7CUS, 7CJR	PAS and TPR-like	β-lactam antibiotics	*V. parahaemolyticus*	[55,56]
VxrA	7LA6, 7KB7, 7KB3, 7KB9	PAS and TPR-like	β-lactam antibiotics	*V. cholerae*	[57]

* Indicates the ligand whose binding site is determined or predicted to be located in the extracellular domain.

PDC ligand-binding sites are not only present in conserved clefts. PhoQ is the HK of the PhoQ/PhoP TCS, which plays an important role in the regulation of virulence in gram negative bacteria such as *Salmonella* [58]. PhoQ is a multisensing kinase that responds to multiple signals, such as divalent cations, low pH, osmotic upshifts, and cationic antimicrobial peptides [58]. A comparison between PhoQ-PDC and CitA-PDC domains shows that the former has the addition of two α-helices and an absence of the cleft region [27,28]. This additional region is located proximal to the lipid bilayer and contains an acidic cluster of amino acids (EDDDDAE). Miller’s group proposed the following model of ligand binding and its activation mode in this region based on structural and biochemical experiments [27,59]. The binding of a divalent cation to this region causes the periplasmic domain of PhoQ to be tethered to the membrane, and PhoQ forms an inactive conformation. In contrast, the replacement of the divalent ion by a cationic antimicrobial peptide eliminates the interaction between the membrane and the periplasmic domain of PhoQ by the divalent cation, resulting in charge repulsion between the membrane and the periplasmic domain of PhoQ. This converts PhoQ into an activated state. Such interactions between membranes and periplasmic domains may occur in other HKs, but they have not yet been reported. In addition, DeGrado’s group proposed that the sensor domain of PhoQ transitions between two conformational states depends on the active state and that the α-helices at the dimer interface move in a scissor-like motion, causing the acidic clusters to be embedded in the membrane. However, the molecular mechanism by which various ligands that bind to acidic clusters contribute to the dynamic displacement of the sensor domain is not clear [60].

PhoQ also contains other signal-sensing regions in the periplasmic domain. In *E. coli*, PhoQ is activated by the small membrane protein SafA [61,62]. Since the expression of SafA (65 amino acids, aa), which is a type II (single pass and C-terminus domain located in the periplasm) transmembrane protein, is induced by another TCS (EvgS/EvgA), SafA serves as a connector between these two TCSs. SafA directly interacts with the sensor domain of PhoQ at its C-terminal region (25 aa) and enhances the kinase activity of PhoQ [63]. Although it was first assumed that SafA also interacted with the acidic cluster of PhoQ, SafA was capable of enhancing PhoQ activity in an acidic cluster neutralized mutant (QNNNNAQ) [64], which was blind to divalent cations or cationic antimicrobial peptides [62]. A series of site-directed mutagenesis studies found that SafA could not activate a PhoQ D179R mutant due to its loss of physical interaction with the sensor [62]. Furthermore, the crystal structure of the sensor domain of this D179R mutant shows a loss of a cavity that is formed by the core β-sheet of the PDC and the N-terminal α1 helix in wild-type PhoQ [34]. The presence of this cavity is assumed to be important for the interaction with SafA, although the molecular mechanism remains unclear.

UgtL, which is present in *Salmonella enterica*, is a functional homolog of SafA [65]. This inner membrane protein with two TM helices and a 24-amino acid periplasmic region also activates PhoQ via direct interaction. The expression of UgtL is positively regulated by the PhoQ/PhoP system and by a different TCS: SpiR/SsrB [66]. PhoQ activation by UgtL is involved in the pathogenicity of *Salmonella* in mice, which requires sensing of mildly acidic pH by PhoQ. Similar to SafA, UgtL promotes PhoQ autophosphorylation. Whereas the periplasmic region of SafA is sufficient for PhoQ activation, UgtL requires both its periplasmic and transmembrane regions to activate PhoQ [65]. In contrast, PhoQ activity is repressed by another small membrane protein, MgrB [67,68,69,70]. Similar to UgtL, the periplasmic, TM, and cytoplasmic domains are all involved in the interaction between PhoQ and MgrB (more details are provided in Section 3).

In the case of EnvZ HK, which responds to the shift in osmotic pressure and regulates the outer membrane protein with its cognate OmpR RR, the PDC domain binds CHAPS detergent in the V-shaped hydrophobic cavity formed by the β-sheet and α-helices (non-canonical binding site of PDC) [36]. CHAPS is structurally similar to sodium cholate, a bile acid component that is abundant in the intestine. When sodium cholate is applied to *E. coli* cells, OmpC expression, which is positively regulated by EnvZ/OmpR, is suppressed. This suggests that EnvZ/OmpR may be involved in the stress response to bile acids. In addition, EnvZ is activated by MzrA, a membrane protein whose expression is regulated by the CpxA/CpxR TCS [37,71]. MzrA, similar to SafA, has a region in the periplasm that is necessary for EnvZ activation, but its detailed mechanism of action is not clear. In addition, CusS HK, which senses copper in the periplasm and is involved in the regulation of Cu^2+^ efflux pump expression, has four Ag(I): two Ag(I) are located in the dimer interface (intermolecular binding) formed by the α helix, and one Ag(I) is located in each molecule of the same α helix (intramolecular binding) [38]. Furthermore, in the quorum-sensing LuxQ HK, signal autoinducer-2 (AI-2) does not bind directly to the tandemly arranged PDCs but interacts with another periplasmic protein, LuxP, which forms a complex with LuxQ. LuxQ recognizes AI-2 via LuxP for activation [32,43]. Thus, the same PDC structures of HKs have different signals and sensing regions.

#### 2.1.2. Sensing by an All α-Helix Type Structure

Another representative structural class that has been studied as well as PDCs is the all α-helix type, which, as the name suggests, consists of only α-helices. This structure was first identified in the chemotaxis receptors Tar and Tsr [72], and later in NarX, a HK that senses nitrate/nitrite and is involved in the control of anaerobic respiration [44]. The periplasmic regions of NarX and another nitrate/nitrite-sensing NarQ HK have a common motif, P-box (17aa). Amino acid mutations in the P-box change the response to nitrate, suggesting that this region is involved in ligand binding [73]. Structural analysis revealed that the α-helix containing the P-box forms a homodimer, and a single ligand binds to the P-box at the dimer interface [44]. This binding mode is different from that of Tar and Tsr, which have multiple ligand-binding sites [74].

Similar to NarX, TorS is also known to be involved in anaerobic respiration and has an all-α-helix type structure [48]. The periplasmic structure of TorS consists of two four-helix bundles stacked on top of each other. This four-helix bundle was also observed in NarX and NarQ. The four-helix bundles of NarX and NarQ can be neatly superimposed on the four-helix bundle domain proximal to the membrane of TorS. However, as is the case of the aforementioned PDC class, the sequence homology is low, even though the structures are similar. In addition, the membrane-anchored protein apo-type (ligand-unbound) TorC inhibits TorS activity by binding to the periplasmic region of TorS [75]. The detailed mechanism of TorC binding and inhibition of TorS activity is unknown.

**Figure 2:**
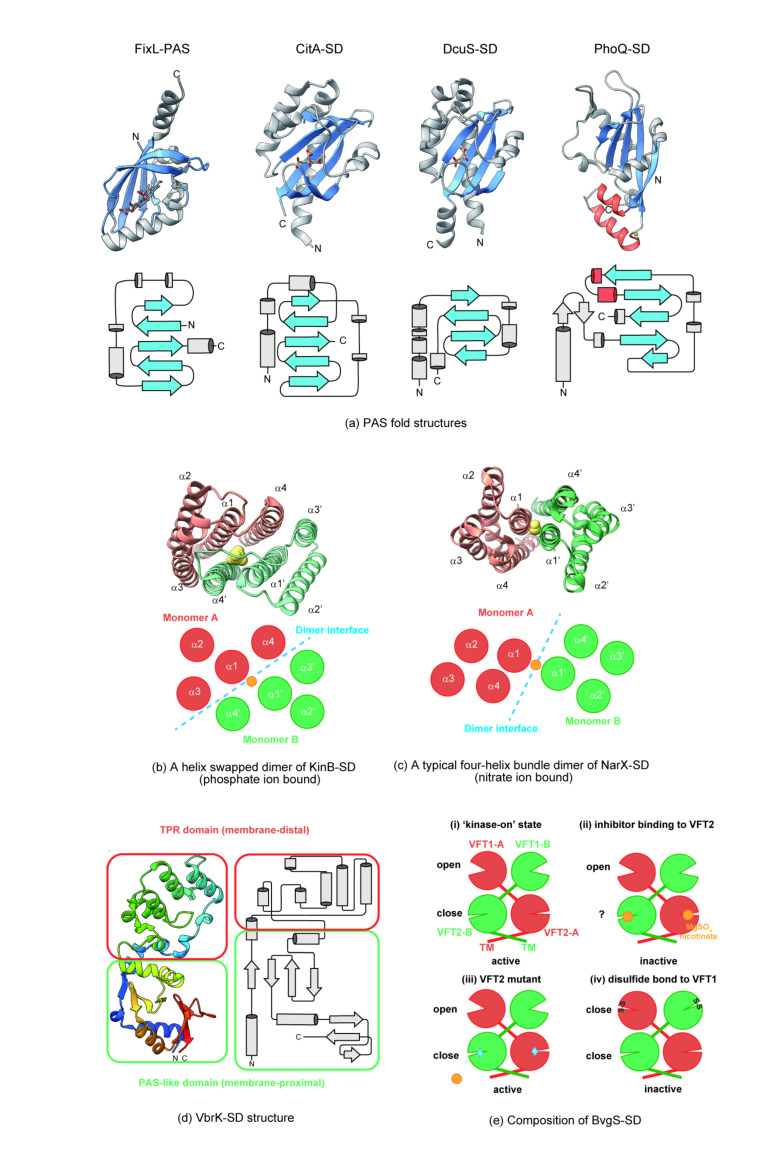
Structure comparison of extracellular sensor domains. (**a**) Comparison of PAS fold structures. FixL (1DP6) has a PAS fold as an intracellular signal-sensing domain. CitA (2J80), DcuS (3BY8), and PhoQ (3BQ8) have a PAS fold as an extracellular signal-sensing domain. The β-strand indicated in blue is a conserved core structure in the PAS fold, and signal molecules are bound to this region in FixL (ferric heme), CitA (citrate), and DcuS (L-malate), whereas in PhoQ, two α-helices (red) containing acidic clusters are present in addition to the PAS-fold. A nickel ion (green sphere) is bound to the additional helix. (**b**,**c**) Comparison of the dimer interface of the all α-helix type-sensing domains [50]. The upper panels show top-down view of KinB (3KKB) and NarX (3EZH), while the lower panels show schematic representation of the α-helix arrangement. The ligand molecules, which are phosphate ion for KinB and nitrate ion for NarX, are shown in yellow. The dimer interface of KinB is formed by six helices, while that of NarX is formed by two helices (blue dashed line). (**d**) Representative secondary and ribbon diagrams of VbrK-SD(7CUS). The TPR-like region is located at the distal side of the membrane and the PAS-like region is located at the proximal side of the membrane in VbrK-SD. (**e**) Configurations of the VFT (Venus flytrap) structure of the BvgS homodimer and its kinase activity. (i) Kinase-active state with no ligand binding to the VFTs. (ii) Kinase-inactive state with the inhibitor, nicotinate or MgSO_4_ (yellow sphere), binding to the VFT2. Whether VFT2 is in a closed or open state under this condition is not clarified. (iii) Kinase-active state in which the inhibitor cannot bind to the VFT2 mutant with a mutation at its ligand binding site. (iv) Kinase-inactive state in which the VFT1 is artificially closed with disulfide bonds by Cys mutations.

KinB HK, which acts as a negative regulator of alginate biosynthesis in *Pseudomonas aeruginosa*, has a unique four-helix bundle assembly. KinB has an all-α-helix-type structure in the periplasm and forms dimers but exchanges the fourth α-helix (α4) between molecules to form a two-symmetrical dimer (Figure 2b) [50]. The dimeric interface of the KinB sensor is formed by a total of six α-helices by swapping α4 intermolecularly. It differs from NarX, which assembles a dimer with two α-helices forming the dimer interface (Figure 2b,c). At the dimeric interface, the Arg side chain, which originally extends toward the dimer interface, is bent outward by the Asp side chain of the neighboring α-helix, forming a cavity that can accommodate a 200–300 Da molecule. This cavity is thought to be the binding site for signal molecules [50].

#### 2.1.3. Sensing by Other Structures

Some periplasmic sensor domains have unique structures and ligand-binding modes other than PDCs and all α-helix types. The BvgS/BvgA TCS of *Bordetella pertussis* activates virulence-activated genes (vags), which are critical factors in infection [76]. Jacob-Dubuisson’s group reported that the periplasmic region of BvgS HK has Venus flytrap (VFT, PBPb; bacterial periplasmic solute-binding protein) domains in tandem (Figure 2e) [52,53]. This region of BvgS forms a homodimer in the form of two molecules crossing each other. Generally, a VFT domain is a two-lobe domain that closes the open structure in response to a specific ligand. VFT1, located distal to the membrane, has an open structure. In contrast, VFT2, located proximal to the membrane, has a closed structure, even without a ligand. BvgS is stably active in this closed state of VFT2 but becomes inactivated when an inhibitor (such as nicotinate or MgSO_4_) binds to VFT2. In addition, amino acid mutations in the ligand-binding region of VFT2 cause BvgS to become constantly active. However, the activity of BvgS is lost when the open structure of VFT1 (distal to the membrane) is artificially closed by Cys cross-linking. Thus, the structural state of the two unique domains in the periplasm of BvgS contributes to its activity.

Furthermore, a new sensor structure has recently been reported. *Vibrio parahaemolyticus*, a major foodborne pathogen causing seafood-associated intestinal inflammation, produces β-lactamases and is resistant to β-lactam antibiotics. Production of this β-lactamase is regulated by the VbrK/VbrR TCS [77]. The periplasmic region of VbrK consists of two heterogeneous domains: the domain distal to the membrane is identified as tetratricopeptide (TPR)-like and the proximal domain as PAS-like based on their structural homology (Figure 2d) [55,56]. The PAS-like domain has no conserved cleft. In contrast, a pocket was observed in the TPR-like domain, which is assumed to be a potential candidate for the binding site of the ligand β-lactam antibiotic [55]. However, this has not yet been experimentally clarified. In contrast, Goh et al. performed an interaction analysis between β-lactams and VbrK using isothermal titration calorimetry and reported that there was no interaction between the two [56]. Since VbrK has a TPR-like domain, they assume that a β-lactam antibiotic-sensing protein binds to this domain and activates the VbrK/VbrR system. VxrA HK of *Vibrio cholerae*, which is homologous to VbrK, is also involved in resistance to β-lactam antibiotics [57]. The crystal structure of the sensor domain of VxrA has been shown to adopt two different packing conformations, suggesting that this conformational change is involved in signal transduction to the cytoplasm. Similar to VbrK, cavities are observed at the membrane-distal site, but it is unclear whether these are the binding sites for the substrates [57].

### 2.2. How the Signals Are Transduced from Periplasmic Sensors to the Cytoplasmic Domain

The signal received at the periplasmic region needs to be transmitted to the cytoplasmic domain through the transmembrane region. This signaling mechanism is considered to be relatively universal because of structural similarities (most TM domains form four-helix bundles). Although conformational changes between the structures of the activated and inactivated states of full-length membrane-bound HKs will be informative for understanding how the signal is transmitted, no such structure has been solved to date. However, several molecular mechanisms have been proposed to explain the transmission of the signal through the membrane.

The mechanism of signal transduction from the periplasmic region to the cytoplasmic region has been well studied in the chemoreceptor aspartate receptor (Tar). It has been shown that ligand binding to the periplasmic region causes a piston-like downward movement of one transmembrane helix relative to the other helix [78]. The same piston-like movement has also been reported for HKs. In the nitrate-sensing NarX, phosphorylation is enhanced by an upward movement of the transmembrane region upon ligand binding, in contrast with Tar [38,43,77].

Piston-like movements have also been reported in NarQ and TorS, which have structures highly homologous to NarX [17,48,49]. The relationship between the movement of the TM and the phosphorylation activity is correlated: dephosphorylation occurs when TM2 is pulled toward the cytoplasm, and phosphorylation occurs when TM2 is pulled toward the periplasm. Moreover, in CitA, citrate binding to the periplasm causes domain contraction, which pulls the C-terminal β-strand, resulting in piston movement of the TM helices [79]. DcuS, which has a structurally identical PDC, is also suggested to move similarly to CitA, based on biochemical experimental approaches [80].

In contrast, Bayesian modeling of PhoQ, based on the results of cross-linking experiments with Cys introduced into the TM region, suggests that the signal is transmitted by a scissor-like movement [60]. This model proposes that ligand binding to the periplasmic region induces a scissor-like movement, which leads to dynamic rearrangement of the TM helices and signal transduction to the cytoplasm. In this model, there is no piston-like movement in the TM helices of PhoQ. A similar scissor-like movement has also been reported in hybrid HK BT4663 of *Bacteroides thetaiotaomicron* [54].

In LuxQ HK, an asymmetric LuxQ dimer is formed upon signal binding, and the rotation of the TM helix of this dimer transmits the signal across the membrane to the cytoplasmic domain [32]. In addition, HptS, a G6P sensor from *Staphylococcus aureus*, undergoes a conformational change when G6P binds to the periplasmic protein HptA, which forms a complex with HptS. It has been proposed that the movement is transmitted to the cytoplasmic region by the rotation of the second TM, activating the system [42].

Although several signaling models have been proposed, it is not clear whether these are common to all sensor HKs or are unique to each HK. Gordeliy’s group considers their model, which was proposed based on their latest crystal structure and molecular dynamics analysis of NarQ [17], as “simply different degrees of freedom in coiled-coil proteins” and state that it can occur in any HK [45].

## 3. Transmembrane Sensing and Control

Some membrane-anchored HKs lack periplasmic or extracytoplasmic sensor domains (Figure 1c). These HKs are mostly controlled by stimuli perceived at their TM domains and are introduced as “intramembrane-sensing HKs” (reviewed in [18,81]). Accumulating information shows that even HKs with extracytoplasmic sensor domains can sense additional signals in their TM domain. These HKs integrate information from different domains for a fine-tuned response.

### 3.1. Intramolecular Sensing

**Figure 3:**
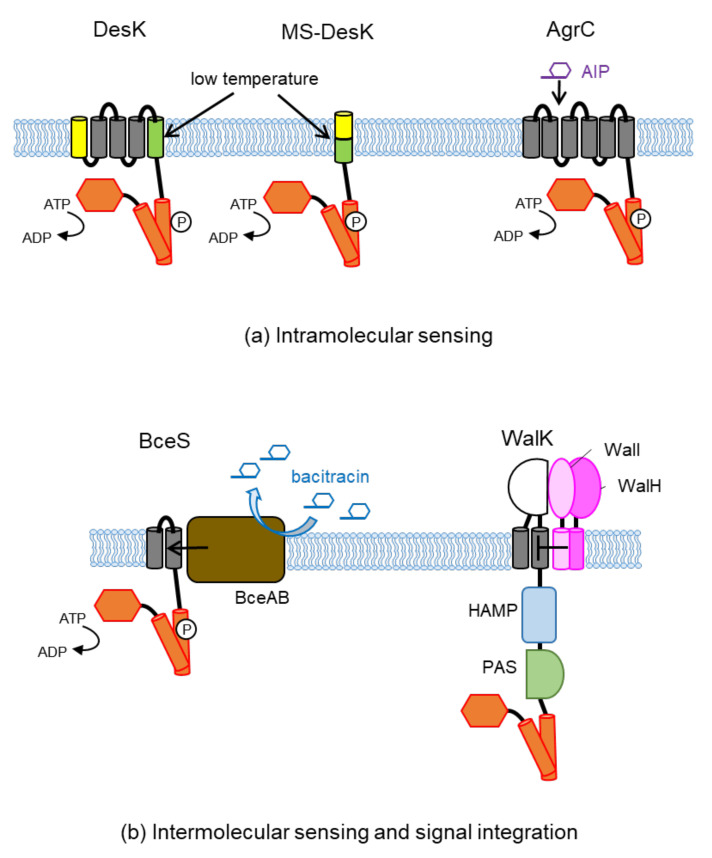
Signal perception in the TM domain. Modulating signals can be sensed either directly (**a**) or indirectly via other proteins (**b**). (**a**) DesK and MS-DesK (minimal sensor-DesK, which has only a single chimera TM helix of the N-terminal half of TM1 and the C-terminal half of TM5) sense low temperature by detecting the changes in membrane thickness, fluidity, and water permeability [86]. AgrC is modulated by the direct binding of a quorum-sensing factor, AIP (autoinducing peptide) [87]. (**b**) BceS forms a sensory complex with an ABC transporter, BceAB. The flux of peptide antibiotics such as bacitracin activates BceS [88]. Although WalK has both extracellular and intracellular signal domains, WalK activity is also modulated by two membrane proteins, WalH and WalI, through interaction at the TM domain. Whereas WalH and WalI repress WalK activity in *Bacillus subtilis* [89], these two proteins activate WalK in *S. aureus* [90,91]. HKs normally exist as dimers but are shown as monomers for simplification.

DesK is a membrane-bound HK in *B. subtilis* that has five transmembrane helices but no extracytoplasmic sensor domains (Figure 1c). Together with its RR, DesR, this TCS responds to low temperature (below 28 °C) and induces the expression of its target, the *des* gene, which codes for a Δ5 desaturase [82,83]. The desaturase inserts into the membrane and catalyzes the introduction of cis-double bonds into acyl chains of phospholipids to restore membrane fluidity [83]. DesK detects the change in temperature in the TM domain (Figure 3). Among the five TM helices, TM1 and TM5 are considered to play key roles in temperature sensing. Since membrane proteins with multiple TM helices are technically difficult to handle in biochemical studies, a truncated version of DesK, MS-DesK (minimal sensor-DesK), which only has a single membrane-spanning α helix (chimera of the N-terminal half of TM1 and the C-terminal half of TM5) and the cytoplasmic catalytic domain (DesKC) (Figure 3), has been employed in many studies. MS-DesK responds to a cold signal in a way that is similar to the full-length DesK [84]. Individual expression of TM1-DesKC or TM5-DesKC cannot respond to temperature changes, but coexpression of TM1-DesKC and TM5-DesKC enables detection of the change in temperature [85]. This also supports the view that TM1 and TM5 are the key players in sensing cold signals.

Structures of the cytoplasmic portion (DesKC) in phosphatase and kinase active states have been determined [92]. A comparison of these two structures suggested that the temperature signal into DesKC is transduced via the rotation and tilting of the two helices that enter the DHp from its N-terminal side [12]. It is proposed that DesK detects changes in membrane thickness, fluidity, and water permeability because of temperature variations. Molecular mechanisms for detecting such changes in the TM domain have been investigated biochemically and through MD simulations and are summarized into the following three determinants of thermodetection (DOTs) [86].

DOT1 (sunken buoy [93]): The Phe8/Lys10 located at the N-terminus of TM1 anchors the TM helix to the extracellular water–lipid interphase and provides a piston-like motion to TM1 in response to the membrane thickness.

DOT2 (serine zipper [94]): Three serine residues located on the same face of TM5 form hydrogen bonds with water due to increased membrane hydration at high temperatures. However, at low temperatures, the membrane becomes less hydrated, and the serine hydroxyl groups form hydrogen bonds with the serines in the TM5 of the other protomer, creating a serine zipper.

DOT3 (dual linker [95]): The positively charged region that connects TM5 with the cytoplasmic catalytic domain adopts a helix/coil conformational duality triggered by membrane thickness (random coil interaction with the membrane at high temperature; helix is detached from the membrane at low temperature), modulating the kinase state.

DOT1 plays an important role in sensing temperature variation, but DOT2 plays a predominant role and serves as the master activation switch. Another key factor is the dynamics around the two conserved proline residues (Pro16 in TM1 and Pro148 in TM5), where the first Pro16 acts as a hinge and the second Pro148 acts as a fulcrum around which TM5 can pivot, producing scissor-like motions that are presumably translated as rotations to the subsequent domain. TM1 is involved in sensing membrane lipid fluidity/thickness, and by interacting with TM5, it transmits information to the catalytic domain associated with TM5 [12].

PhoQ HK in *E. coli* and *Salmonella* responds to changes in the membrane. As described in the previous section, the periplasmic sensor domain of PhoQ senses divalent cations, acidic pH, and antimicrobial peptides [58]. In addition to these signals, Yuan et al. found that an osmotic upshift activates the PhoQ/PhoP system [96]. Interestingly, a PhoQ mutant lacking the periplasmic sensor domain still responds to osmolarity, indicating that the sensor domain is not essential for osmosensing. Deletion studies and MD simulations suggest that osmosensing of PhoQ relies on a conformational change within the TM domain induced by a perturbation in cell membrane thickness and lateral pressure under hyperosmotic conditions [96].

AgrC is an example of transmembrane sensing via ligand binding. This HK regulates virulence via quorum sensing in *S. aureus *[87]. AgrC contains six TM helices without extracytoplasmic domains (Figure 3). A macrocyclic peptide pheromone named the autoinducing peptide (AIP) binds to the TM domain of AgrC (possibly to the extracellular loops 1 and/or 2; [97,98]) and activates this HK. Upon binding of the AIP, the interdomain linker, which connects the TM domain and the kinase domain, rotates and thus promotes kinase activity [99]. Structural analysis data of the AIP–AgrC complex are not available at present. However, 3-D solution-phase structures of *S. epidermidis* AIPs and AIP analogs from NMR spectroscopy have been reported, providing new insights into AIP–AgrC interactions [100].

Similarly, in *Enterococcus faecalis*, a gelatinase biosynthesis-activating pheromone (GBAP; a small autoinducing lactone-linked cyclic peptide of 11 amino acid residues) is proposed to bind to the extracytoplasmic region of FsrC HK. Binding of GBAP activates the FsrC/FsrA TCS and induces the expression of virulence factors, such as GelE gelatinase and SprE serine protease [101,102].

### 3.2. Intermolecular Signal Transfer

Signal detection in the TM domain can be performed indirectly via another protein that serves as the direct sensor of the signal. In this case, the HKs themselves could not detect their stimuli in the absence of their accessory proteins. Protein partners involved in intramembrane-sensing HKs were reviewed by Mascher [81]. In some cases, these accessory proteins are known as transporters. HKs and transporters form complexes in the membrane.

The presence of accessory proteins often represses HK activity. However, in *B. subtilis* BceS, as well as in other Bce-like systems found in many gram-positive bacteria, HKs fully rely on their cognate transporters for activation and remain inactive when the transporters are absent [103]. BceS has only two TM helices and lacks an extracytoplasmic domain (Figure 3). This HK forms a sensory complex with BceAB, an ATP-binding cassette (ABC) transporter, which mediates resistance against bacitracin and several other antimicrobial peptides, possibly by pumping out these peptides. The expression of the *bceAB* transporter operon is regulated by the BceSR TCS. BceS responds to peptide antibiotics, such as bacitracin, via their associated BceAB ABC transporter. By combining system biological modeling with in vivo experiments, Fritz et al. showed that the activity of the transporter (or the flux of antimicrobial peptides) controls BceS activity, which then induces *bceAB* expression [88]. They proposed the BceSR–BseAB system as a “flux-sensor”. Bce-like systems respond to many antimicrobial peptides and confer antimicrobial resistance to various gram-positive species, including important pathogens such as *S. aureus* and *E. faecalis*. The “flux-sensor” is recognized as a novel sensing system for detecting antimicrobial substances [88]. The molecular mechanisms by which the activity of the transporter BceAB controls BceS activity remain unclear. However, in a recent cross-linking and modeling study, BceS was shown to be activated via helical rotations in the DHp domain, accompanied by a piston motion in the second TM helix [104].

Structural analyses of the TM domain of intramembrane-sensing HKs have been reported for NsaS (also called BraS and BceS) in *S. aureus* [105]. The structure of the short N-terminal amphiphilic helix, two TM helices, and the intracellular linker connecting the TM domain to the cytoplasmic catalytic domains have been described using a combination of solution state NMR spectroscopy, chemical cross-linking, and MD simulations. The cytoplasmic linker is a marginally stable α helical coiled coil, and the antibiotic-induced signal proceeds by coiled-coil conformational switching in the linker [105].

Some HKs are fused to transporters. The *Ks*Amt5 HK of an anaerobic ammonium-oxidizing bacterium, *Candidatus* Kuenenia stuttgartiensis, combines a membrane-integral ammonium transporter domain with a fused histidine kinase. Although the ammonium transporter (Amt) domain conserves the known structural features of Amt transporters, it does not efficiently translocate NH_4_^+^ across the membrane. Instead, the Amt domain shows high selectivity for NH_4_^+^ and specifically binds to the ion. This binding elicits a conformational change in the Amt domain, which is transduced intramolecularly to modulate kinase activity [106].

### 3.3. Intramolecular Signal Integration

Most of the aforementioned transmembrane-sensing HKs lack extracytoplasmic sensor domains and detect their signals in the TM domain. In contrast, as described in the previous section, a prototypical HK senses the signal at its sensor domain and transmits this information to the cytoplasmic catalytic domain via the TM domain. The two TM helices of the TM domain transmit signals by motions such as piston-like, rotational, and scissor-like [10]. When these conformational changes are modified by accessory proteins, the activity of HKs is altered and is usually repressed. Thus, information from the extracytoplasmic sensor and TM domains is integrated into the TM domain. This integrated information is then transmitted to the downstream domains [18]. These accessory proteins are membrane proteins with TM domains involved in interaction with their target HKs. Whether or not these accessory proteins sense signals such as the transporters in Bce-like systems described in the previous section is mostly unknown. In some cases, their expression levels control the activity of their target HK.

An example of modulating signal conversion at the TM domain is the WalH and WalI (formerly YycH and YycI) membrane proteins that control the activity of WalK (formerly YycG) HK in *B. subtilis* [89]. WalK is the HK of the WalK/WalR TCS, which regulates cell wall turnover and cell division, making this system essential for cell growth. This TCS is conserved by low G+C gram-positive bacteria, including *S. aureus* and *Streptococcus pneumoniae*. WalH (455 aa) and WalI (280 aa) are located in the extracytoplasm and are tethered to the membrane via a single N-terminal TM helix. They directly interact with WalK and repress its activity by forming a complex assembled through their TM helices (Figure 3). Both the TM helices of WalH and WalI are required to modulate the activity of WalK, but their large extracytoplasmic domains are unnecessary [89,107]. Thus, WalH and WalI interact and modulate WalK activity in their TM domains. The function of the extracellular domains of WalH and WalI remains unknown. These domains may respond to additional signals to control the WalK activity.

In *S. aureus*, however, WalH and WalI do not play a major role in the negative control of WalK, despite their complex formation with WalK [90,91]. Instead, a membrane protein SpdC with eight predicted TM helices and the Abi domain (Pfam [108] 02517) represses WalK activity by direct binding. SpdC and WalK form a complex and colocalize at the division septum of a cell. The TM domain of SpdC is necessary for interaction with WalK. Since the expression of *spdC* is positively regulated by WalKR, repression of WalK by SpdC forms a negative feedback loop. RNA-seq analysis revealed that SpdC controls the expression of approximately 100 genes, including genes that are directly involved in bacterial virulence upon infection. The SpdC-regulated genes included a number of WalKR regulons. However, there were also many genes whose expression was not regulated by the WalKR system. Instead, the expression of some of these genes was regulated by other TCSs such as SaeSR, VraSR, ArlSR, GraSR, and BraSR. In fact, SpdC was shown to directly interact with nine other HKs in addition to WalK using bacterial two-hybrid (BACTH) assays. When focused on the virulence-related SaeS HK, SpdC directly interacted with SaeS through TM domain contacts and positively regulated the SaeSR TCS [109]. A similar function of a bacterial Abi-domain protein is seen in group B *Streptococcus*. Abx1, a membrane protein with eight predicted TM helices and an Abi-domain, also directly interacts with CovS HK in the TM domain. Abx1 negatively regulates CovS, leading to the activation of virulence gene expression [110].

Interaction between TM helices is also necessary for the *Salmonella* and *E. coli* membrane protein, MgrB, to modulate the activity of PhoQ HK. The PhoQ/PhoP TCS is critical for virulence in various gram negative pathogens, such as *Salmonella* and *K. pneumonia* [58]. PhoQ/PhoP directly regulates *mgrB* expression. MgrB, which is a small membrane protein of 47 amino acids, directly interacts with PhoQ to repress its activity, forming a negative-feedback loop [67,68]. Inhibition of PhoQ requires the TM domain of MgrB. Trp20 within the TM domain is the key residue for PhoQ/MgrB complex formation [69]. However, in the case of MgrB, the short cytoplasmic N-terminal and periplasmic regions are also involved in the repression of PhoQ activity. Furthermore, MgrB responds to the periplasmic redox state via its two conserved periplasmic cysteines [70], adding more input signals that modulate PhoQ.

Bacterial small proteins of less than 50 amino acids encoded by small open reading frames have been predicted and identified. Several small proteins, such as MgrB, have been associated with diverse functions (reviewed in [111,112]), but the function of most of the small proteins remains unknown. Many of these bacterial small proteins are hydrophobic with a single TM domain and are localized in the membrane, as is the case with MgrB. Interactions between TM helices of these small proteins and their target membrane proteins (some of them may be HKs) can be predicted [113] and await further investigation.

## 4. Cytoplasmic Sensing

Signals in the cytoplasm are mostly perceived via sensing domains and/or at the catalytic domain (reviewed in [19]). The sensing domains are usually PAS or GAF domains. The HAMP domains that are found in nearly 31% of HKs are essential for signal transduction [114], although they are not recognized to sense signals. The main function of HAMP is assumed to be in converting signals from the TM helices to those that can be recognized by the downstream cytoplasmic domains. This conversion can be diagonal scissoring, helical rotation, and transitions between stable and dynamic states [13,115,116,117,118]. The activity of the catalytic domain is modified by the binding of accessory proteins and second messengers. Some studies have proposed that this domain can also directly sense signals. In this section, we introduce the wide diversity in signal recognition by the various cytoplasmic domains, as well as the recently recognized additional signal sensing occurring in the cytoplasm.

### 4.1. Sensing at the Helical Linker

**Figure 4:**
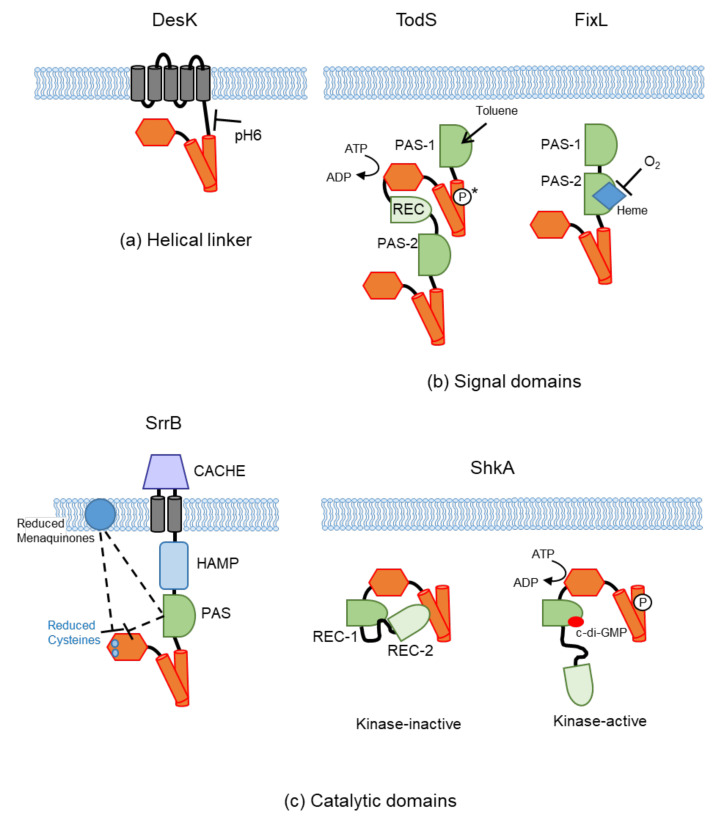
Signal perception in the cytoplasm. Examples of signal perception in different cytoplasmic domains are shown. (**a**) Helical linker. DesK senses acidic pH by destabilization of the helix that links the TM domain with the DHp domain [121]. (**b**) Signal domains. TodS is activated by the binding of toluene to the first PAS domain (* it is not clear whether autophosphorylation occurs at the first or the second kinase domain) [122]. FixL is inactivated by binding of O_2_ to heme, which is a cofactor in the second PAS domain of FixL [123]. (**c**) Catalytic domains. SrrB is inactivated by a reduced condition. The redox sensitive cysteines in the CA domain respond to reduced menaquinones either directly or indirectly through the PAS domain [124]. SrrB has an extracellular CACHE (calcium channels and chemotaxis receptors) domain. ShkA is inactivated in the absence of c-di-GMP because ShkA adopts a compact domain arrangement that is kinase-inactive. When c-di-GMP binds to the pseudoreceiver domain (REC-1), the canonical REC-2 domain is liberated and changes ShkA into a kinase-active state [125].

The parallel 2-helix coiled coil linker that connects different domains to the DHp domain is present in many HKs and is called the signaling helix (S-helix) [119]. A similar linker that connects the TM domain with the DHp domain is also found in the temperature-sensing DesK and plays a crucial role in controlling HK activity [120]. According to Bortolotti et al., the membrane-proximal region of this linker is involved in pH sensing by DesK (Figure 4) [121]. The kinase activity of DesK is activated below 27 °C but is repressed at pH6 even below 27 °C. The acidic pH causes the protonation of glutamate residues located close to the lipid membrane. This breaks the salt bridges and destabilizes the helix, resulting in signaling blockage. When DesK activity is repressed at low pH, membrane lipid fluidity decreases, which may restrict the entrance of substrates, conferring low pH fitness to the cell [121].

### 4.2. Sensing at the Intracellular Signal Domains (PAS and GAF)

Nearly 33% of HKs contain PAS domains, and about 9% contain GAF domains [114]. Other minor sensing domains also modulate HK activity (Table 2). PAS sensors are found in all kingdoms of life, and these sensors detect chemical and physical stimuli. The structures and signaling mechanisms of PAS domains have been extensively reviewed by Möglich et al. [16]. The GAF domains share similar structures with the PAS domains [126]. We introduce examples of a wide range of signals sensed in the cytoplasmic PAS and GAF domains.

#### 4.2.1. Ligand Binding

Similar to the periplasmic PAS (PDC) domains of CitA and DcuS, which bind their ligands, the cytoplasmic TodS HK binds to its signal, toluene, at its PAS domain for TodS kinase activation [122]. The TodS/TodT TCS in *P. putida* regulates the expression of genes involved in the degradation of aromatic hydrocarbons into Krebs cycle intermediates via the toluene dioxygenase pathway. Toluene binds to the N-terminal PAS domain of the two PAS domains in TodS (Figure 4) [122]. Another example is zinc binding to the cytoplasmic PAS domain of WalK HK of *S. aureus*. WalK consists of an extracellular PAS, TM domain, HAMP domain, cytoplasmic PAS domain, and catalytic domain. Analysis of a suppressor mutant of WalK/WalR activity revealed a tyrosine substitution (H271Y) in the cytoplasmic PAS domain of WalK. Structural analysis of the cytoplasmic PAS domain revealed a metal-binding site, in which Zn^2+^ was tetrahedrally coordinated by four amino acids, including His 271. Exogenous Zn^2+^ concentration affected the expression of WalR-controlled genes, showing that the cytoplasmic PAS served as an intracellular sensor that activates the WalK HK via interaction with Zn^2+^ ions [127].

#### 4.2.2. Cofactor Binding

Several PAS domains bind to cofactors for signal perception (Table 2). The cytoplasmic PAS domains of HKs mostly bind to heme or flavine adenine dinucleotide (FAD) to sense the redox state. For example, the FixL HK of nitrogen-fixing rhizobia is a heme-based oxygen sensor. FixL is composed of a C-terminal histidine kinase domain and N-terminal tandem PAS domains (PAS-A and PAS-B, Figure 1c). The second PAS domain (PAS-B) senses O_2_ through the heme cofactor (Figure 4) [123]. O_2_ binds to heme and strongly represses FixL kinase activity, whereas CO acts as a weak inhibitor of FixL. Crystal structures of deoxy-and CO-bound FixL heme-PAS domains have been reported [128].

FixL of *Bradyrhizobium japonicum* is a soluble cytoplasmic HK without TM helices, and the structure of full-length FixL has been determined together with its RR, FixJ [129]. Comparison of active and inactive forms of FixL showed that intramolecular signal transduction is driven by local changes in the PAS domain and the coiled-coil region connecting the PAS-B and catalytic domains.

Another cofactor is FAD, which is housed in the PAS-A domain of MmoS HK in the methanotroph *Methylococcus capsulatus* [130]. MmoS functions in copper-mediated regulation of soluble methane monooxygenase. Methane monooxygenases catalyze the oxidation of methane to methanol and are potentially useful for bioremediation. The crystal structures of MmoS tandem PAS domains, PAS-A and PAS-B, were determined, and the FAD cofactor was housed solely within PAS-A. The redox state is proposed to be perceived by FAD and mediates the copper switch [148]. Furthermore, O_2_ is bound to the [4Fe-4S]^2+^ cluster in the PAS domain of NreB HK in *S. carnosus* [131].

Cofactors also bind to GAF domains. DosT HK of *Mycobacterium tuberculosis* consists of two tandem GAF domains whose N-terminal heme-binding GAF domain directly binds O_2_, NO, and CO. O_2_ dissociation from the N-terminal GAF domain initiates autophosphorylation via the C-terminal kinase domain. The crystal structures of the active oxygen-free and inactive oxygen-bound states of the N-terminal GAF domain are available [132].

#### 4.2.3. Others

The redox state is also sensed by disulfide formation within the PAS domain. The ArcB/AcrA system in *E. coli* is controlled by oxygen availability and regulates the transcription of genes involved in oxidative and fermentative catabolism. ArcB HK has a short periplasmic sequence of only 16 amino acids between the two TM helices, and a cytoplasmic PAS domain resides between the second TM helix and the atypical catalytic domain, which consists of DHp, CA, REC, and HPt domains. Georgillis et al. reported that quinones affect ArcB activity [133]. Under aerobic growth, ubiquinone electron carriers silence ArcB via intermolecular disulfide bond formation at Cys180 and Cys241 [149]. Upon a shift from aerobic to anaerobic growth conditions, menaquinonols reduce cysteine residues for ArcB activation [150,151,152].

BvgS HK of *B. pertussis* (the whooping cough agent) and EvgS HK of *E. coli* have cytoplasmic domain structures that are similar to ArcB, a cytoplasmic PAS domain between the TM helix and the atypical catalytic domain consisting of DHp, CA, REC, and Hpt domains. Although ArcB has only a short periplasmic sequence, both BvgS and EvgS have large periplasmic domains, which are composed of two tandem bacterial periplasmic solute-binding protein (PBPb) domains. These periplasmic domains are necessary for perceiving the stimuli for modulating HK activity: nicotinate and sulfate ions for negative regulation against BvgS and acidic pH for positive regulation against EvgS [52,153,154]. To clarify the function of the cytoplasmic PAS domain, Bock and Gross examined the effect of quinones on the kinase activities of the cytoplasmic soluble forms of BvgS and EvgS and found that oxidized ubiquinone also inhibited the kinase activities of BvgS and EvgS [155].

However, in contrast with ArcB, which is positively regulated under anaerobic growth conditions, EvgS can only be activated by acidic pH under aerobic growth conditions. Growth under anaerobic conditions inhibited EvgS activity. A mutant strain with a defect in ubiquinone biosynthesis was not able to activate EvgS at acidic pH, possibly through the involvement of Cys671 within the PAS domain [134]. This result contradicts the in vitro results of oxidized ubiquinone inhibiting EvgS kinase activity [155]. The in vitro study was performed using a truncated cytoplasmic region of EvgS, and the results of in vitro studies may not necessarily reflect the actual cell response. Emphasis on employing full-length HKs in in vitro studies has been recently reviewed [156]. Although the molecular mechanism of how ubiquinone and ubiquinol control EvgS remains unclear, this may be an example of the integration of two signals in the PAS domain [134].

The function of the BvgS PAS domain has been extensively examined, but its function remains unknown. Dupré et al. concluded that the major function of the PAS domain is to maintain the conformational tension imposed by the periplasmic moiety of BvgS [157]. A recent study suggested that the BvgS PAS domain may function as an independent signal perception domain, as shown by in vivo animal experiments using *B. bronchiseptica* strains with mutations in the PAS domain [158].

### 4.3. Sensing at the Catalytic Domain

HK activity is also modulated in the catalytic domain. It has been reported that accessory proteins binding to the DHp domain and phosphatases against phosphotransfer proteins control HK activity. Recent reports suggest the involvement of second messengers and even direct sensing of changes in the cytoplasmic environment. We introduce examples of modulation of HK activity in the catalytic domain.

#### 4.3.1. Protein Binding

The *B. subtilis* KinA and KinB HKs play essential roles in the initiation of sporulation. Two proteins, Sda (46 aa) and KipI (240 aa), are known to inhibit the kinase activity of KinA: KipI against KinA and Sda against both KinA and KinB. Low-resolution structural models of the KinA-Sda and KinA-KipI complexes were determined using data from small-angle X-ray scattering and small-angle neutron contrast variation. Both Sda and KipI have been proposed to bind between the conserved autophosphorylated histidine residue and the hairpin bend of the four-helix bundle [137,138]. The interacting surface on KinA is wider for KipI compared with Sda because of its larger size. Hence, it has been suggested that KipI sterically blocks interactions between the DHp and CA domains, whereas Sda has been suggested to cause an allosteric effect by inducing a conformational change in the four-helix bundle [138]. The crystal structure of *Geobacillus stearothermophilus* KinB with Sda has confirmed Sda binding to the base of the DHp domain, with axes of Sda helices roughly perpendicular to the KinB DHp helices [139]. Based on this structure and biochemical experiments, Bick et al. argued that Sda inhibits KinB autokinase activity by sterically blocking the interactions between the DHp and CA domains, similar to the inhibition mechanism of KipI suggested by Jacques et al. [137,139].

Another protein that binds the DHp domain is PtsN, which stimulates the activity of KdpD HK in *E. coli* [140]. The KdpD/KdpE system maintains K^+^ homeostasis by controlling the expression of the *kdpFABC* operon, which encodes a high-affinity K^+^ transporter [159]. PtsN, a member of the phosphoenolpyruvate-dependent phosphotransferase system that operates in parallel with the canonical sugar transport phosphotransferase (PTS^Ntr^), changes its phosphorylation state by nitrogen and carbohydrate availability. Only non-phosphorylated PtsN can efficiently bind KdpD for activation, suggesting that PTS^Ntr^ coordinates K^+^ uptake with the metabolic state of the cell [140]. Through BACTH, surface plasmon resonance, and ligand fishing experiments, Mörk-Mörkenstein et al. have shown that PtsN specifically binds to the DHp domain of KdpD and forms a PtsN/KdpD_2_/KdpE ternary complex. The proposed model is that when a non-phosphorylated PtsN binds to a protomer of a KdpD dimer, it stimulates autophosphorylation of the other protomer [141]. High amounts of non-phosphorylated PtsN lead to the binding of PtsN to both protomers, which outcompetes KdpE and reduces the level of phosphorylated KdpE [141]. Non-phosphorylated PtsN also binds to and activates another HK, PhoR, which controls the phosphatase starvation response [160].

#### 4.3.2. Second Messengers

Cyclic dinucleotides (CDNs), which are newly recognized second messengers, are enzymatically synthesized from two nucleotides within the cell and serve as signaling molecules that control various physiological processes. In bacteria, CDNs are involved in the control of cellular processes such as motility, virulence, biofilm formation, and cell cycle progression [161]. Among CDNs, cyclic di-GMP (c-di-GMP) is the most widespread and has been studied in bacteria. Recently, the involvement of c-di-GMPs in controlling the activity of HKs has been reported.

*Caulobacter crescentus*, a bacterium with an asymmetric division cycle, employs the CckA/ChpT/CtrA system (ChpT is the phosphotransfer protein, CtrA is the RR) to control its cell cycle. C-di-GMP directly binds to CckA HK to inhibit kinase activity and stimulate phosphatase activity. This allows replication initiation and cell cycle progression, which drives the cell cycle [162]. The crystal structure of the CA domain in complex with c-di-GMP and AMPPNP Mg^2+^ was determined by Dubey et al. [146]. A monomeric c-di-GMP molecule binds to the edge of the β-sheet in the CckA CA domain and reciprocally regulates kinase and phosphatase activity through noncovalent cross-linking of the CA domain with the DHp domain. In a different study, Mann et al. showed that CckA uses a cytoplasmic tandem PAS domain sensor to integrate two different signals [135]. Using CckA reconstituted on liposomes, they showed that the first PAS domain (PAS-A) responded to surface CckA density, and the second PAS domain (PAS-B) interacted with c-di-GMP, resulting in stimulation of CckA phosphatase activity. Because only half of c-di-GMP is bound to the CA domain in the crystal structure, c-di-GMP is suggested to lie at the interface between PAS-B and the CA domain [135,146].

ShkA, a cytosolic hybrid HK, is another kinase that is involved in the regulation of *C. crescentus* cell cycle progression [163]. In contrast with CckA, ShkA is activated by increasing levels of c-di-GMP. Dubey et al. solved the crystal structure of the pseudoreceiver domain (REC1) in complex with c-di-GMP. Further NMR and biochemical studies revealed that this c-di-GMP binding leads to liberation of the canonical REC2 domain from the other domains, thereby changing the HK into a dynamic, multiconformational state that enables the phosphoryl transfer reactions required for activation [125] (Figure 4).

It has also been reported that c-di-GMP specifically binds to RavS HK of the phytopathogenic gram negative bacterium *Xanthomonas campestris* and stimulates the kinase activity of RavS. From molecular docking of c-di-GMP to the DHp-CA structure of RavS, which was predicted by homology modeling, it was suggested that c-di-GMP also binds to the CA domain of RavS [147]. Other CDNs also modulate HK activity. Moscoso et al. showed that c-di-AMP binds to the cytoplasmic universal stress protein (USP) domain of *S. aureus* KdpD HK and inhibits the upregulation of the *kdpFABC* operon under salt stress [136].

#### 4.3.3. Others

Redox-sensitive cysteines are found not only in the PAS domain but also in the CA domain. The *S. aureus* SrrAB system is a global regulator of virulence and promotes resistance to nitrosative stress and hypoxia, which is found at sites of infection [164]. By examining the SrrB DHp-CA crystal structure, Tiwari et al. found an intramolecular cysteine disulfide bond in the SrrB CA domain [124]. The presence of this disulfide bond affected biofilm formation, toxic shock syndrome toxin-1 production, and infection in a rabbit model. SrrAB has been reported to respond to reduced menaquinones [164,165], and the proposed model of Tiwari et al. is that the reduced menaquinone pool acts directly on the cysteine residues in the CA domain, or indirectly through the cytoplasmic PAS domain that is located proximal to the membrane [124] (Figure 4).

In some HKs, the cytoplasmic domain can directly sense stimuli from changes in the cytoplasmic environment. This is exemplified in detail by the osmosensing and pH sensing of EnvZ HK in *Salmonella* and *E. coli* (reviewed in [166]). The EnvZ/OmpR system is one of the best-characterized TCSs that regulates the expression of outer membrane porins OmpF and OmpC in response to osmotic stress [167]. EnvZ consists of a periplasmic sensor domain, TM domain, HAMP domain, and catalytic domain of the DHp and CA domains. The cytoplasmic C-terminus of EnvZ (180–450, consisting of DHp and CA domains and a portion of the HAMP domain) is capable of sensing osmolarity and pH in vivo without being inserted into the membrane. By amide hydrogen-deuterium exchange mass spectrometry analyses, Wang et al. found that osmolytes, such as NaCl and sucrose, promoted intrahelical H-bonding, enhancing helix stabilization and increasing autophosphorylation [142]. The stabilized helix included the His^243^ autophosphorylation site and the OmpR-binding site. This stable conformation facilitates increased autophosphorylation and phosphotransfer to OmpR. The osmosensing core has been narrowed down to a 17-amino acid region (238–254) containing His^243^.

The change in intracellular pH is also sensed by the cytoplasmic region of EnvZ [143,144]. Sensing acidic pH in the cytoplasmic region has also been reported for *Salmonella* PhoQ HK [169]. Another study using combined NMR and crystallography found a pH-mediated conformational change involving a sidechain rotameric switch of the catalytic histidine residue that inactivates the phosphatase activity of *Thermotoga maritima* HK853 HK. This study confirmed that the same pH-sensing mechanism is also present in *Salmonella* EnvZ [145]. Ghosh et al. further reported that the stabilization of a disordered backbone helix, induced by osmolality or pH change, leads to changes in the microenvironment of the catalytic His^243^ in *Salmonella* EnvZ [170]. This results in enhanced autophosphorylation by relieving the inhibition and repositioning of the side chain of Asp^244^ and imidazole rotamerization of His^243^.

However, Mideros-Mora et al. are opposed to the pH-gated conformational switch. They have found that the rotamer disposition of the catalytic His is not influenced by the environmental pH from structural studies of HK853-RR468 and analyses of HK structures extracted from the PDB. Instead, they propose that the acidic pH (below pH6) changes the catalytic His into a protonated form, thereby reducing its nucleophilic capacity to catalyze the autophosphorylation and phosphatase reaction [145]. The catalytic domain is highly conserved among HKs. It is not known whether this direct sensing at the catalytic domain is common among HKs or whether it distinguishes the subtle changes in the amino acid sequence or structure of the catalytic domain.

## 5. Concluding Remarks

In the present article, we introduce some examples of how HKs can perceive multiple signals in various domains. Although we are aware that we have ignored many other cases, such as oligomerization and heterooligomerization of HKs and cross talk between noncognate RRs [171], we hope that our examples represent the diversity in how HKs control their activity. A well-studied PhoQ HK senses divalent cations, acidic pH, cationic antimicrobial peptides, and small membrane proteins in its periplasmic domain, osmolarity in its TM domain, and acidic pH in its cytoplasmic domain. Similarly, KdpD HK, which has also been studied in depth, senses K^+^ concentration extracellularly and intracellularly [172] and is also controlled at its cytoplasmic domain via accessory proteins and second messengers. We assume that this multisensing ability should also be applied to other less-studied HKs. Future investigations to understand the underlying mechanism of how HKs sense and integrate their signals will contribute to the development of drugs targeting HKs, as well as their application in bioremediation and food production.

## Figures and Tables

**Table 2:** Examples of cytoplasmic-sensing domains and their signals.

Sensor	PDB	Sensing Domain	Signal/Ligand	Organism	Ref.
DesK		helical linker	pH	*B. subtilis*	[121]
TodS		PAS	toluene	*Pseudomonas putida*	[122]
WalK	4MN5, 6	PAS	Zn^2+^	*S. aureus*	[127]
FixL	1BV5, 6 1XJ2, 3, 4, 6	PAS + heme	O_2_	*B. japonicum*	[24,128,129]
MmoS		PAS + FAD	O_2_	*Methylococcus capsulatus*	[130]
NreB		PAS + [4Fe-4S]^2+^cluster	O_2_	*S. carnosus*	[131]
DosT	2VZW	GAF + heme	O_2_	*M. tuberculosis*	[132]
ArcB		PAS	redox	*E. coli*	[133]
EvgS		PAS	redox	*E. coli*	[134]
CckA		PAS	c-di-GMP	*Caulobacter crescetus*	[135]
ShkA	6QRJ, 6QRL	REC	c-di-GMP	*C. crescetus*	[125]
KdpD		USP	c-di-AMP	*S. aureus*	[136]
KinA	2ZP2	DHp	KipI	*B. subtilis*	[137]
KinA, KinB		DHp	Sda	*B. subtilis*	[138,139]
KdpD		DHp	PtsN	*E. coli*	[140,141]
EnvZ		DHp	Osmolality, pH	*Salmonella, E. coli*	[142,143]
HK853	5UHT, 6AZR 6RGY, 6RFV, 6RGZ, 6RH0	DHp	pH	*T. maritima*	[144,145]
SrrB	6PAJ	CA	redox	*S. aureus*	[124]
CckA	5IDM	CA	c-di-GMP	*C. crescetus*	[146]
RavS		CA	c-di-GMP	*X. capestris*	[147]
